# Fabrication of Biaxially Aligned Nanofibers via Insulating Block‐Assisted Electrospinning and Their Applications

**DOI:** 10.1002/marc.202400888

**Published:** 2025-02-20

**Authors:** Yogita M. Shirke, Dae Hwan Kang, Kwang Won Kim, Byungil Hwang, Song Jun Doh, Ki Ro Yoon

**Affiliations:** ^1^ Textile Innovation R&D Department Korea Institute of Industrial Technology 143, Hanggaulro, Sangnok‐gu Ansan‐si Gyeonggi‐do 15588 Republic of Korea; ^2^ School of Integrative Engineering Chung‐Ang University Seoul 06974 Republic of Korea; ^3^ HYU‐KITECH Joint Department Hanyang University 222, Wangsimni‐ro, Seongdong‐gu Seoul 04763 Republic of Korea

**Keywords:** biaxial alignment, electrospinning, energy and environmental applications, insulating blocks, nanofiber

## Abstract

Electrospinning is a well‐established and widely adopted process for producing fine and continuous nanofiber networks. Electrospun nanofibers have gained significant attention owing to their advantages, including nanoscale fiber uniformity, tunable pore size with bimodal distribution, and versatility in integrating various inorganic and organic compositions. Recently, considerable efforts have been made to align nanofibers and enhance their functionality with improved mechanical properties, faster charge transport, and more efficient mass transport in well‐organized spatial structures. This mini‐review highlights the fabrication of precisely aligned nanofibers using insulating block‐assisted electrospinning. By manipulating the electric field between the nozzle and substrate, combined with a moving substrate, insulating block‐assisted electrospinning enables the biaxial alignment of the nanofibers. This review discusses recent advancements in insulating block‐assisted alignment techniques and explores the applications of these aligned nanofibers in the environmental and energy fields, including air filtration media, lithium‐ion battery electrodes, hybrid gel polymer electrolytes for aqueous batteries, and reinforced composite membranes for fuel cells. In addition, the perspectives associated with the extension of insulating block‐driven aligned nanofiber applications to a wide range of fields and industries is summarized.

## Graphical abstract

This mini‐review presents insulating block‐assisted electrospinning as a novel technique for fabricating biaxially aligned nanofibers. Precise control of the whipping motion of the charged jet enables high‐quality alignment and enhances the performance of nanofiber mats. Furthermore, recent applications in energy and environmental fields are explored, highlighting the potential of this approach for advanced nanofiber technologies.

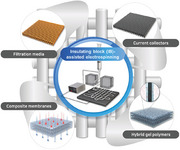

## 1 Introduction

Nanofibers have gained significant attention in recent decades owing to their diverse applications in environmental fields, energy conversion and storage, tissue engineering, food packaging, and sensor technologies. Compared to conventional materials, nanofibers offer distinctive advantages such as a high surface‐to‐volume ratio, bi‐modal pore size distribution, high porosity, adaptable surface properties, and superior mechanical characteristics.^[^ ^1^, ^2^, ^3^, ^4^, ^5^, ^6^, ^7^, ^8^ ^]^ Among the various fabrication methods, electrospinning (e‐spinning) is one of the most widely used because of its simplicity, scalability, versatility, and compatibility with a wide range of materials.^[^ ^9^ ^]^ Over the past 20 years, advances in e‐spinning have increasingly focused on the controlled production of well‐aligned and highly ordered nanofiber networks. These structures exhibit enhanced properties such as improved physicochemical properties including mechanical strength, higher tensile ratios or sensitivity, faster mass or charge transport, and optical or thermal benefits, making them ideal for specific applications.^[^ ^10^, ^11^, ^12^, ^13^, ^14^, ^15^, ^16^ ^]^

During the e‐spinning process, a charged polymeric solution forms a Taylor cone at the nozzle and is subsequently ejected, elongating into sub‐micron fiber jets under the influence of an electric field (E‐field). However, the random whipping motion of the fiber jets, caused by electrostatic repulsive forces, typically results in a nonwoven mat with randomly networked fibers. To address this limitation, alignment methods for e‐spun fibers can be broadly categorized into two main approaches: elimination or utilization of the whipping motion.

Elimination methods suppress or reduce the whipping motion to achieve aligned fibers. These methods include rotating collectors, where high‐speed rotating drums or disks align fibers mechanically,^[^ ^17^ ^]^ the incorporation of centrifugal force to guide fibers radially outward,^[^ ^18^ ^]^ shortening the tip‐to‐collector distance to minimize instability,^[^ ^19^ ^]^ and near‐field e‐spinning, which deposits fibers directly with high precision at short working distances.^[^ ^20^ ^]^

In contrast, utilization methods utilize the whipping motion of the electrospun jet to achieve fiber alignment by strategically manipulating the surrounding electrostatic environment. The utilization methods include the use of auxiliary solid templates to guide fiber deposition,^[^ ^15^ ^]^ patterned collectors to create localized E‐fields for alignment,^[^ ^21^, ^22^ ^]^ and parallel or grating‐like electrodes to steer the jets during flight.^[^ ^23^ ^]^ Additional approaches include U‐shape collectors for path‐specific alignment,^[^ ^24^ ^]^ dielectric materials for modifying the E‐field,^[^ ^25^ ^]^ and rotational methods for dynamically adjusting the jet trajectory.^[^ ^26^, ^27^ ^]^ Post‐treatment methods, such as mechanical stretching or thermal annealing, can further refine the alignment.^[^ ^28^, ^29^ ^]^

Despite these advancements, most alignment techniques focus primarily on uniaxial arrangements, limiting their ability to create complex grid‐like structures or control interfiber porosity. Biaxially aligned nanofibers have been achieved through two key techniques: patterned collector e‐spinning and insulating block (IB)‐assisted e‐spinning, both of which utilize E‐field manipulation to achieve alignment. Patterned collector e‐spinning utilizes conducting electrodes with various pattern geometries to form localized E‐fields, facilitating fiber alignment via electrostatic templates created by previously deposited fibers.^[^ ^30^, ^31^ ^]^ However, this method heavily depends on the dielectric properties of the fibers. For prolonged production times, the electrostatic template induced by the fibers deposited on the patterned collector is degraded, leading to a reduction in the alignment quality.^[^ ^32^ ^]^ This process may also require post‐transfer steps to deposit aligned fibers on the desired surface, adding complexity and limiting scalability.

In contrast, IB‐assisted e‐spinning modifies the E‐filed using insulating blocks (IBs), enabling consistent fiber alignment during the flight of the e‐spun jet.^[^ ^33^ ^]^ Unlike patterned collector methods, IB‐assisted e‐spinning does not rely on substrate‐based electrostatic templates, thus ensuring alignment even during prolonged production. Once deposited on the collector, the conductive fibers effectively dissipate their charges, preventing electrostatic interference between the deposited fibers and the incoming charged jet. Furthermore, this technique enables the fabrication of biaxially aligned nanofiber mats by controlling substrate movement, providing a continuous and versatile solution for creating complex nanofiber architectures.

By addressing the limitations of the conventional techniques, IB‐assisted e‐spinning offers a promising pathway for the development of high‐performance nanofiber structures. This technique has been explored for several applications.^[^ ^34^, ^35^, ^36^, ^37^, ^38^, ^39^ ^]^ In this mini‐review, a concise overview of IB‐assisted electrospinning and its applications is provided with the aim of inspiring researchers and manufacturers to further develop and expand the use of aligned nanofibers across various fields.

## 2 IB‐Assisted E‐Spinning

**Figure 1:**
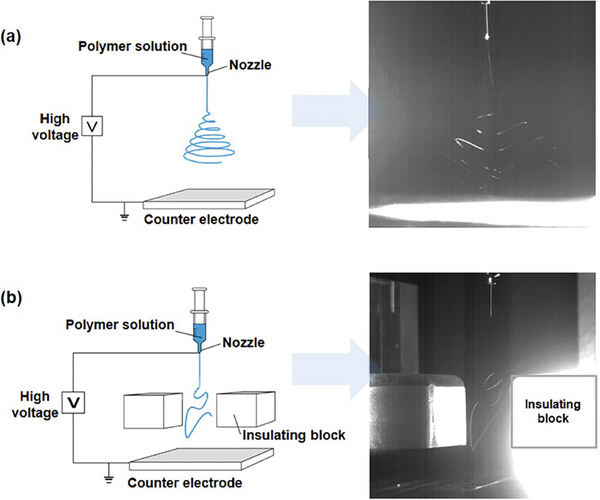
Schematic illustration and photograph images of a) conventional e‐spinning and b) IB‐utilized e‐spinning for the jet using a polymer solution. Reproduced with permission^[^ ^33^ ^]^ Copyright 2023, IOP Science.

E‐spinning is a highly effective and versatile method for fabricating fibrous nanomaterials with large surface areas and high porosity, making it a popular choice for diverse applications.^[^ ^40^, ^41^, ^42^ ^]^ In conventional e‐spinning methods (**Figure** **1**a), a high electrical potential is applied to the nozzle, causing the polymeric solution to be ejected as an electrified jet. As the solvent in the jet evaporates, the polymer nanofibers spiral down onto the grounded substrate, forming an irregularly entangled nonwoven structure with multiple pores between the stacked fibers.^[^ ^43^ ^]^ To address the limitations of conventional e‐spinning in achieving a controlled fiber alignment, Hwang et al. introduced IB‐assisted e‐spinning.^[^ ^33^ ^]^ This method positions two plastic blocks around the nozzle tip, restricting the E‐field along the x‐axis and thereby directing fiber movement primarily in the y‐direction (Figure 1b). When combined with rotating collector motion, this technique can also produce biaxially patterned nanofiber mats. This straightforward setup allows the fabrication of aligned nanofibers with a predetermined orientation, offering a cost‐effective and efficient solution for producing well‐ordered fiber networks.^[^ ^37^ ^]^

Key variables in IB‐assisted e‐spinning, such as the gap between the IBs, nozzle movement speed, collector motion period, distance between the nozzle and substrate, and applied voltage, directly influence the structure and alignment quality of the collected fibers.^[^ ^33^, ^35^, ^37^ ^]^ By carefully adjusting these parameters, researchers can precisely tailor the spatial arrangement and morphology of nanofibers.

**Figure 2:**
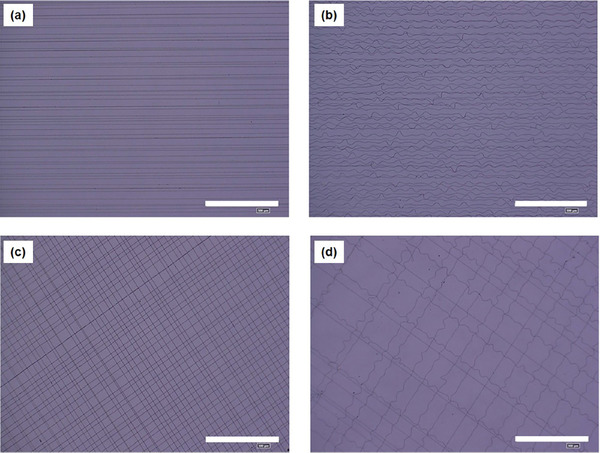
E‐spun polyethylene oxide (PEO) nanofibers display: a) linear, b) wave‐like, c) cross rectilinear shape, and d) cross wave‐like shape configuration. Reproduced with permission.^[^ ^33^ ^]^ Copyright 2023, IOP Science.

For instance, linear or wave‐like patterns can be achieved by adjusting the movement of the nozzle during IB‐assisted e‐spinning, as shown in **Figure** **2**a,b, respectively. The spacing between adjacent nanofibers can be finely tuned by adjusting the speed of the substrate motion along the x‐axis. Faster substrate movement results in a large gap between the fibers, whereas slower motion creates denser fiber arrangements. Furthermore, adjusting the collector angle from 0° to 90° enables the creation of various patterns, such as rectilinear, grid‐like, and slanted alignments (Figure 2c,d), with high reproducibility. Importantly, the applied voltage and IBs gap significantly influenced the resulting fiber structure. Higher voltages and narrower IBs gaps produced rectilinearly aligned nanofibers by increasing the attraction of the charged jet to the collector, thereby restricting the motion of the jet to a defined trajectory. Conversely, lower applied voltages and wider IBs gap allow the jet to oscillate more freely, generating wave‐like fiber patterns. This effect arises from the balance between the E‐field strength and space‐charge interactions. Stronger E‐fields attract the jet more forcefully to the substrate, resulting in narrower and wavier fibers, whereas weaker fields permit greater displacement in the y‐direction, resulting in wider and straighter fibers.

Compared with patterned collector e‐spinning, which relies on electrostatic templates formed by previously deposited fibers, IB‐assisted e‐spinning offers several distinct advantages. First, fiber alignment is minimally affected by the electric field induced by the deposited fibers. This ensures consistent fiber alignment even during prolonged manufacturing processes. Second, IB‐assisted e‐spinning enables the direct deposition of aligned fibers onto the substrate, simplifying the production process and improving scalability. Third, its ability to dynamically control the alignment patterns, including rectilinear and biaxial grid configurations, renders IB‐assisted e‐spinning a more versatile and robust method for creating complex nanofiber architectures. By addressing the limitations of conventional and patterned collector methods, IB‐assisted e‐spinning demonstrates its potential for the scalable, high‐precision fabrication of nanofiber structures tailored for advanced applications in diverse fields.

## 3 Emerging Applications of IB‐Assisted Aligned Nanofibers

**Figure 3:**
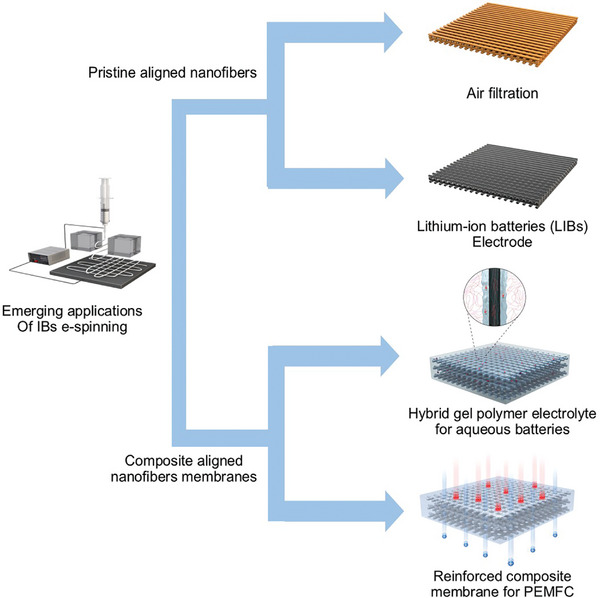
Emerging applications of IB‐assisted e‐spun aligned nanofibers.

**Figure** **3** illustrates recently reported four representative applications of aligned nanofibers fabricated using IB‐assisted techniques.^[^ ^34^, ^37^, ^38^, ^39^ ^]^ The IB‐assisted e‐spinning process is primarily used to produce biaxially aligned nanofibers. These pristine aligned nanofibers could be directly utilized as polymeric nonwoven materials in air filtration media. Carbonized, the aligned carbon nanofibers (CNFs) become effective electrode materials for lithium‐ion battery (LIB) applications. In addition, aligned nanofiber composite membranes, such as hybrid gel polymer electrolytes, can be produced for flexible aqueous batteries, facilitating ionic transport through artificially guided nanofiber channels. Reinforced composite membranes have been developed to enhance the dimensional stability of the polymer electrolyte membranes used in fuel cells. The following sections introduce specific applications and discuss the effectiveness of aligned nanofibers.

### 3.1 Air filtration

**Figure 4:**
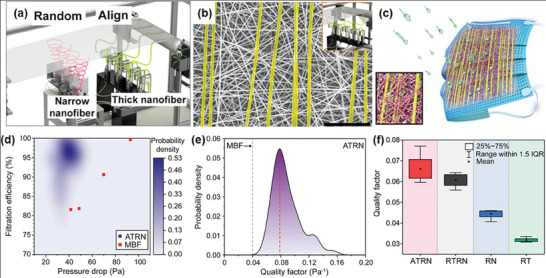
a) Scheme of IB‐assisted e‐spinning for the production of aligned nanofibers; b) SEM image of ATRN; c) diagrammatic representation of the primary filtration mechanisms of ATRN filters in face mask; d) filtration efficiency of ATRN and MB filter, e) Quality factor (QF) of ATRN; and f) assessment of QF as a function of pressure drop for each configuration of the fiber. Reproduced with permission.^[^ ^34^ ^]^ Copyright 2023, American Chemical Society.

Air filters and respiratory mask filters are widely used in various fields, including household environments, health care, and industrial sites, to remove unwanted particles and protect against pollutants.^[^ ^44^ ^]^ Nanofiber nonwoven materials produced by e‐spinning are known for their dense, fine pore structure formed between fibers of uniform thickness, which provides high filtration efficiency through the physical sieving of particles and dust. However, this dense structure can also result in a high pressure drop, making it challenging to achieve both high filtration efficiency and low pressure drop. To address these requirements, Bae et al. developed a multi‐nozzle e‐spinning system incorporating an IB, enabling concurrent control of the fiber alignment and pore structure using a sequential multi‐nozzle e‐spinning setup.^[^ ^34^ ^]^ An E‐field‐guided e‐spinning apparatus within a continuous production system was used to align the nanofibers as shown in **Figure** **4**a.

Using this setup, a partially aligned dual‐nanofiber filter, including a uniaxially aligned thick nanofiber and randomly oriented narrow fiber composite (ATRN), was produced on a roll‐to‐roll collector. In contrast to common randomly collected microfiber membranes, the ATRN exhibited a different diameter distribution, composed of randomly oriented thin (diameter 150 nm) nanofiber base layers and a uniformly aligned thick (diameter 450 nm) nanofiber spacer layer (Figure 4b). The fiber diameter and pore structure could be effectively controlled by controlling the E‐field at the spinning nozzle and regulating the concentration of the polymer solution. In addition to regulating the fiber density and pore‐size distribution, the nanofiber alignment provides sufficient porosity for adjusting the pressure drop and ensuring consistent membrane thickness and sufficient strength of ATRN‐based filters. This configuration and the features of ATRNs are beneficial for various filtration applications, such as face masks, which require a high degree of air permeability for breathing and water resistance (Figure 4c). Unlike conventional melt‐blown filter (MBF), which can degrade when exposed to high moisture levels, ATRN with a physical sieving mechanism maintain their original performance under these conditions.

The filtration efficiency‐pressure drop plot for ATRN compared to conventional MBF is shown in Figure 4d. The configuration of dual nanofibers with submicron pores in ATRN displays increased fiber density and decreased volume density, resulting in a 97% increase in filtration efficiency and a 50% reduction in pressure drop compared to MBF. This demonstrates the ability to control the pore structure and porosity through thickly aligned nanofibers. To verify accurate comparisons of quality factor (QF) with conventional MB filters among different fiber structures, ATRN achieved a record‐high QF value of ≈0.0781, with a mean value of 0.066. This is notably higher than that of the randomly oriented narrow fibers (RN) at 0.045, randomly oriented thick fibers (RT) at 0.031, and randomly oriented thick and narrow fiber composite (RTRN) at 0.061 (Figure 4e,f). This research demonstrates that the introduction of IB‐assisted e‐spinning alignment technology is beneficial not only for developing high‐performance filtration materials, but also for application in protective clothing and gowns.

### 3.2 Lithium‐Ion Batteries Electrode

**Figure 5:**
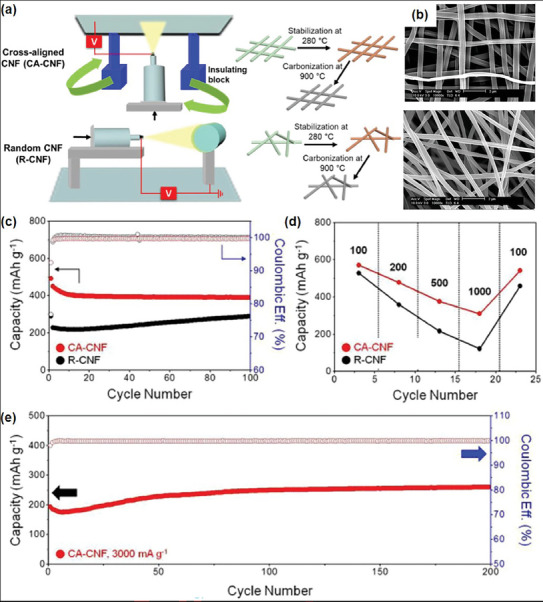
a) Schematic illustration of the fabrication process of CA‐CNF and R‐CNF, b) SEM image of CA‐CNF and R‐CNF, c) cycle retention tests, d) rate capabilities at different current densities of CA‐CNF and R‐CNF, and e) high‐rate cyclability tests of CA‐CNF for 200 cycles. Reproduced with permission.^[^ ^37^ ^]^ Copyright 2021,Elsevier.

Lithium‐ion batteries (LIBs) are one of the most widely adopted energy storage systems, with applications in electric vehicles, laptops, and portable electronics owing to their high energy efficiency, stable operation, and compact and versatile design.^[^ ^45^, ^46^, ^47^, ^48^, ^49^, ^50^ ^]^ LIB cells function through electrochemical reactions between lithium ions and the active materials in the electrodes. However, the limited theoretical capacities of both the anode and cathode, as well as the inactive current collector, which accounts for ≈9.4% of the mass fraction of cells, present challenges for further improvement. To address these limitations, carbon‐based freestanding electrode materials that can serve as both the active materials and current collectors have received considerable attention in recent years.^[^ ^51^, ^52^, ^53^ ^]^ Cheong et al. used cross‐aligned carbon nanofiber (CA‐CNF) via an IB‐assisted e‐spinning.^[^ ^37^ ^]^ Polyacrylonitrile (PAN), a representative precursor for carbon synthesis was dissolved in N,N‐dimethylformamide (DMF) and stirred at 50 °C for 6 h to prepare the e‐spinning solution. Using an IB‐assisted e‐spinning setup, the PAN‐DMF solution was e‐spun under an applied voltage of 10 kV, feed rate of 5 mL min^−1^, and a distance of 10 cm between the syringe tip and the current collector. The biaxially aligned PAN nanofibers were subjected to two heat treatment steps: stabilization at 280 °C for 2 h in air, followed by carbonization at 900 °C for 2 h in an Ar atmosphere. Thus, the biaxially aligned CA‐CNF was successfully fabricated (**Figure** **5**a). In comparison to the randomly networked carbon nanofibers (R‐CNF) produced by conventional e‐spinning (Figure 5b, lower), CA‐CNF exhibited thick deposition with highly perpendicularly aligned fibers (Figure 5b, upper). It is worth noting that the e‐spun solution was conducting, which likely facilitated alignment during prolonged production, as previously discussed.

To evaluate the impact of the CNF alignment in the electrode on the overall cell performance, cycle retention tests were conducted. As shown in Figure 5c, both CA‐CNF and R‐CNF demonstrated similarly high Coulombic efficiencies. However, CA‐CNF exhibited a significantly higher reversible capacity than R‐CNF, along with a more stable capacity retention and reduced variation. In addition, CA‐CNF showed enhanced electrochemical performance at all current densities (100, 200, 500, and 1000 mA g^−1^), with the performance gap between CA‐CNF and R‐CNF becoming more pronounced at higher current densities (Figure 5d). In terms of capacity retention, R‐CNF retained only 23.1% of its initial capacity, whereas CA‐CNF retained 54.2%, representing a 235% higher capacity retention rate. Figure 5e further illustrates the high‐rate cyclability of CA‐CNF performed at a current density of 3000 mA g^−1^. The cell using CA‐CNF maintained a reversible capacity of 259.4 mAh g^−1^ with an excellent coulombic efficiency of 99.9% after 200 cycles. The authors emphasized that the faster kinetics and regular electron pathways provided by the CA‐CNF contributed to superior cycle retention, even at high current densities. This study also provides promising evidence for the broader application of CA‐CNF, functioning simultaneously as both electrodes and current collectors in next‐generation metal‐air batteries.

### 3.3 Hybrid Gel Polymer Electrolyte for Aqueous Batteries

Zinc‐air batteries (ZABs) based on gel polymer electrolytes (GPEs) have emerged as viable candidates for wearable medical tracking devices, fitness domains, entertainment, industry, and military applications, because of their high energy density, safety, flexibility, and cost‐effectiveness.^[^ ^54^, ^55^, ^56^ ^]^ GPE‐based ZABs are a feasible option for wearable power sources and typically incorporate a polymer matrix such as polyvinyl alcohol (PVA), in the form of a hydrogel containing a KOH solution, which provides ionic conductivity and cell flexibility. However, electrolyte evaporation through an air cathode remains a significant challenge, leading to a reduced lifespan and limited practical applications.

**Figure 6:**
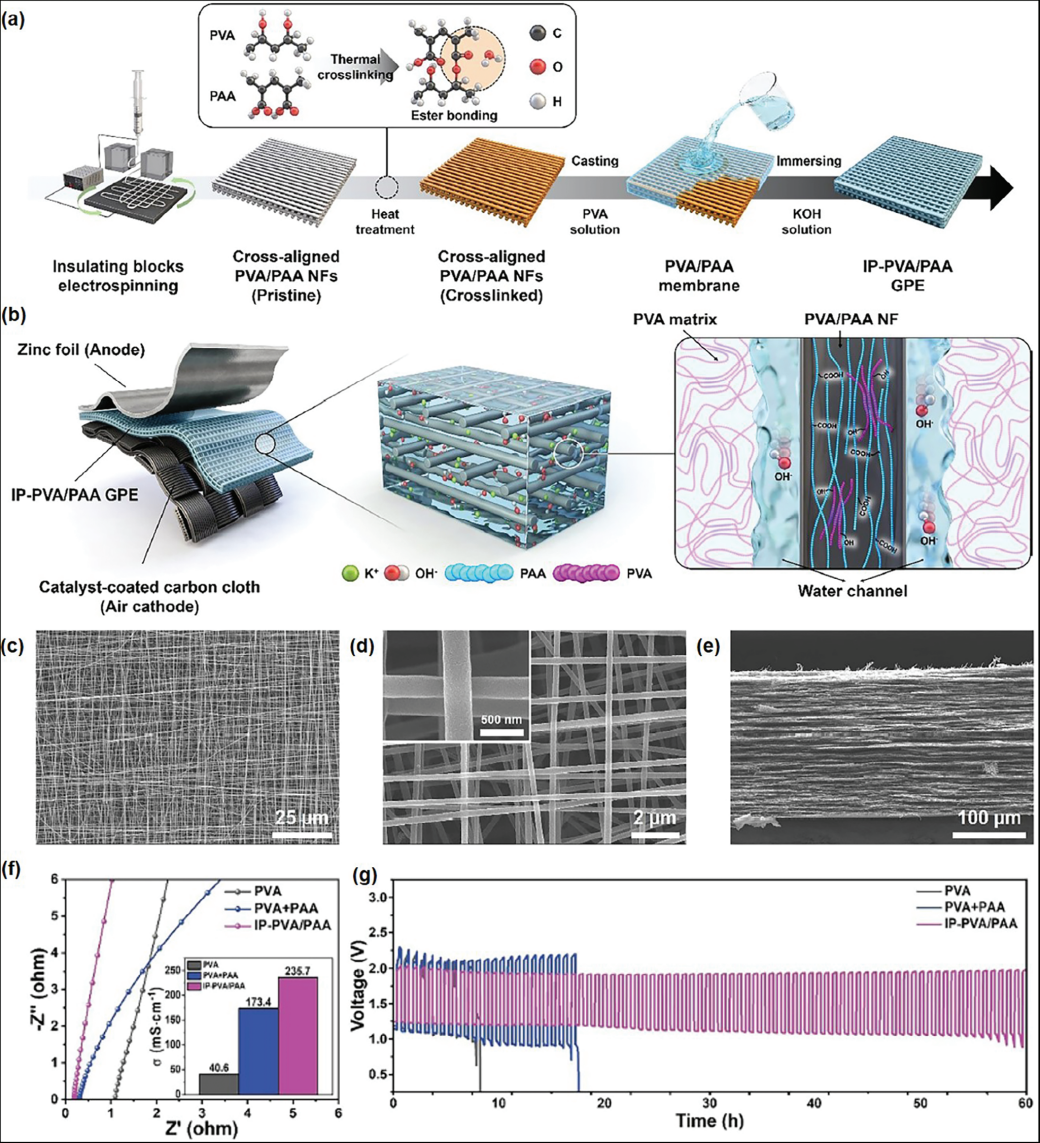
a) Schematic representation of the IP‐PVA/PAA GPE fabrication process, b) sandwich‐type ZAB cell, c) low and d) high magnification SEM images of CA‐NFs, e) cross‐section view of CA‐NFs, f) Nyquist plots and ionic conductivities (inset) of PVA, PVA+PAA, and IP‐PVA/PAA, and g) galvanostatic discharge and charge profiles at 2 mA cm^−2^. Reproduced with permission.^[^ ^38^ ^]^ Copyright 2024, Elsevier.

Kim et al. developed a composite GPE containing polyacrylic acid (PAA)‐based cross‐aligned nanofibers (CA‐NFs) produced by IB‐assisted e‐spinning, with ultra‐hydrophilic CA‐NFs embedded in a PVA matrix (**Figure** **6**a).^[^ ^38^ ^]^ The CA‐NF network was heated at 150 °C for 1.5 h to enhance crosslinking between the alcohol groups in PVA and carboxylic acid groups in PAA, forming ester bonds. The membranes were then fabricated using the doctor blade casting method. Finally, the IP‐PVA/PAA GPE was obtained by dipping the PVA/PAA membrane in a 6 M KOH solution. Upon immersion in 6 M KOH, the superabsorbent PAA unexpectedly formed an internally porous (IP) structure surrounding the aligned PAA/PAA CA‐NFs. Figure 6b shows a sandwich‐type ZAB cell based on IP‐PVA/PAA GPE, where carbon cloth served as the air cathode and the zinc foil as the anode, with the IP‐PVA/PAA GPE situated between them. Owing to the superabsorbent properties of the PAA polymer, the CA‐NFs within the IP‐PVA/PAA GPE could capture a substantial quantity of the liquid electrolyte. This absorption causes the liquid electrolyte to accumulate along the CA‐NFs, forming ideal, internally connected ion transport channels. These channels enable OH^−^ ions to diffuse more quickly than they can through the ion‐hopping mechanism typically observed in pure solid electrolyte matrices. Figure 6c shows a clearly aligned fibrous network formed by the CA‐NFs, with each nanofiber having a mean diameter of 220 nm (Figure 6d). Conducting e‐spinning formulations is likely to provide efficient alignment. E‐spun jets obtained from such formulations carry high charges, as demonstrated by measuring the electric current of the jet, which may facilitate alignment using IB. Consequently, efficient alignment can be ensured by IB e‐spinning, resulting in a thick accumulation of aligned fibers. Thus, the cross‐sectional SEM image reveals well‐stacked e‐spun CA‐NF layers with a thickness of ≈200 µm (Figure 6e). Electrochemical impedance spectroscopy was conducted to measure the ionic conductivities of the membranes (Figure 6f). The inset shows the calculated ionic conductivities of the GPEs based on their x‐axis intercepts. When PAA is blended with PVA, the ionic conductivity increased from 40.6 to 173.4 mS cm^−1^. It is interesting to note that IP‐PVA/PAA exhibited a remarkable ionic conductivity of 235.7 mS cm^−1^, approximately six times higher than pure PVA. The author emphasized that this is among the highest ionic conductivities reported for GPEs in ZABs based on PVA and PAA. Furthermore, the ZAB cell based on IP‐PVA/PAA demonstrated superior cyclic stability and rechargeability for over 80 h (Figure 6g). In contrast, the ZAB cells based on PVA and PVA+PAA showed shorter cycle lives, lasting ≈10 and 23 h, respectively.

This study highlights that IB‐assisted e‐spinning, combined with composite membrane technology and hybrid GPEs, can enhance ionic transport through aligned hydrophilic channels along the CA‐NFs, establishing long‐lasting performance characteristics in aqueous ZABs. This innovative approach supports the commercialization of flexible ZABs and expands their potential applications in the wearable device market.

### 3.4 Porous Reinforcement for Composite Membrane in Fuel Cells

The global demand for hydrogen energy has increased significantly, with fuel cell electric vehicles (FCEVs) emerging as one of the most promising and widespread commercial applications of hydrogen fuel.^[^ ^57^ ^]^ Polymer electrolyte membrane (PEM) fuel cells are commonly used in vehicle applications because of their low operating temperature (<100 °C), suitable power output range, and high portability and efficiency. However, mechanical and chemical degradation of PEM under dynamic vehicle operating conditions continues to hinder the widespread commercialization of FCEVs. To address this issue, reinforced composite membranes (RCMs) composed of an expanded polytetrafluoroethylene (ePTFE) membrane as the mechanical support and perfluorosulfonic acid (PFSA) as the proton‐conductive polymer have attracted considerable interest because of their promising performance in PEM fuel cells. Despite their effectiveness, the preparation of RCMs faces critical challenges owing to immiscible reactions between the hydrophilic sulfonate groups in PFSA and the hydrophobic nanoporous ePTFE matrix.

**Figure 7:**
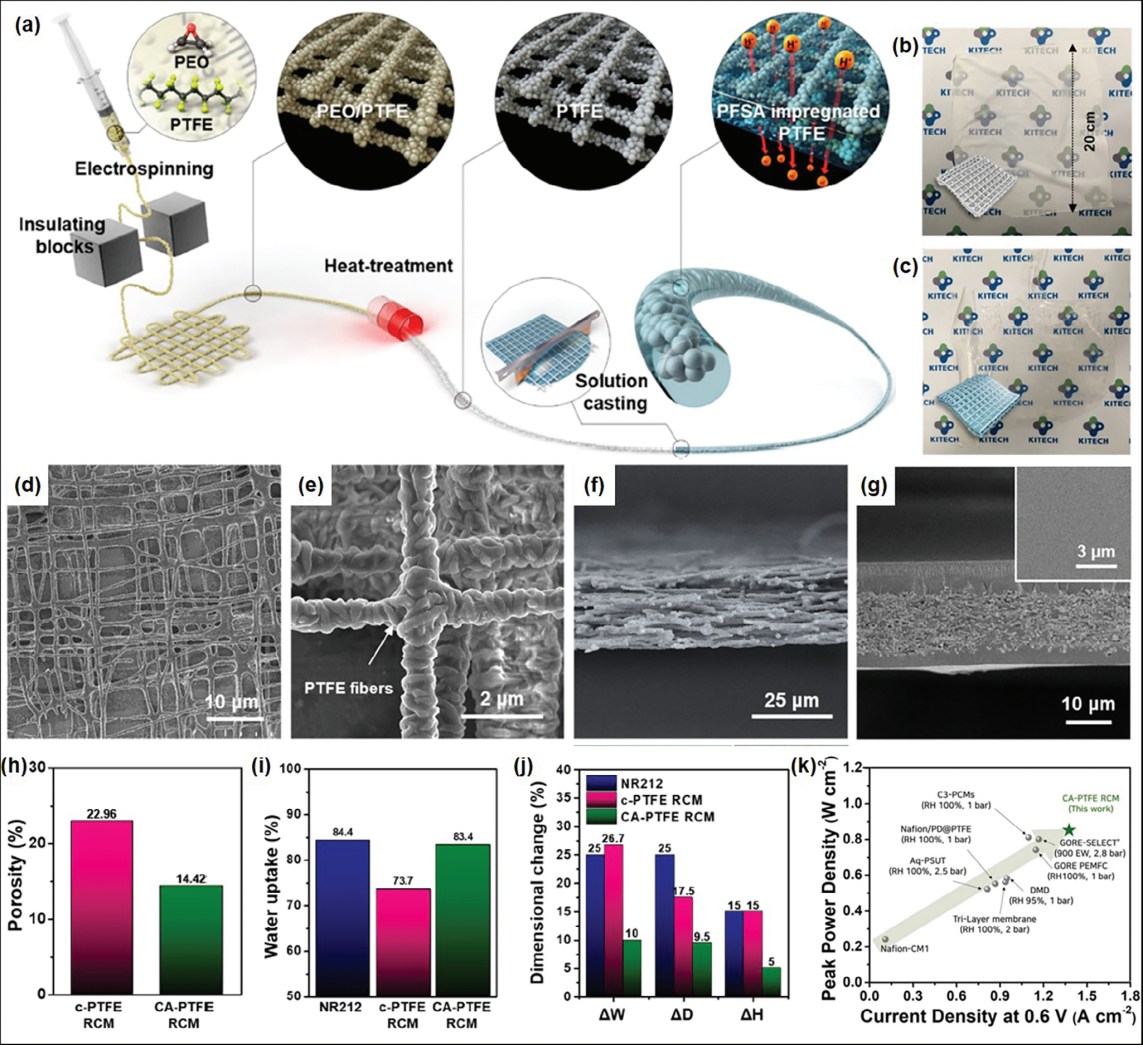
a) Schematic illustration of the synthesis of CA‐PTFE by IB e‐spinning, b) digital photograph images of CA‐PTFE and c) CA‐PTFE RCM, d) low and e) high magnification SEM images of aligned PEO/PTFE fibers, f) cross‐section view of CA‐PTFE, g) cross‐section view of CA‐PTFE RCM, h) porosity values, i) water uptake property, and j) dimensional stability of c‐PTFE RCM and CA‐PTFE RCM, k) hydrogen crossover current densities of CA‐PTFE RCMs. Reproduced with permission.^[^ ^39^ ^]^ Copyright 2022, Elsevier.

Recently, Hwang and Lee et al. proposed that pore size‐ and pattern‐regulated, mechanically robust e‐spun PTFE nanofiber mats as novel reinforcements for high‐performance and durable RCM in fuel cell applications.^[^ ^39^ ^]^ The synthesis of the cross‐aligned PTFE (CA‐PTFE) supports involved two steps. First, PTFE particles were dispersed in polyethylene oxide (PEO), and aligned PEO/PTFE composite fibers were fabricated using an IB‐assisted e‐spinning setup (**Figure** **7**a). Heat treatment was then conducted at 400 °C for 10 min to remove the PEO component, leaving only thermally fused PTFE particles with the cross‐aligned framework. The developed CA‐PTFE support was impregnated with a proton‐conductive PFSA resin solution using the doctor‐blade method, followed by drying the solution‐cast membrane for several hours. The photograph in Figure 7b shows a pristine CA‐PTFE mat, and Figure 7c shows a CA‐PTFE‐based RCM that appeared nearly transparent after casting with the PFSA ionomer.

The perpendicularly aligned morphology of CA‐PTFE was clearly observed in the SEM image (Figure 7d) of the as‐spun aligned PEO/PTFE fibers obtained from the E‐field guided e‐spinning process. The SEM images clearly show well‐aligned fibers forming an orthogonal framework. After thermally removing the PEO matrix covering the PTFE particles in the fibers, the remaining PTFE particles merged partially, yielding a fibrous structure with micron‐scale pores (Figure 7e). Cross‐sectional SEM image confirmed that the thickness of the CA‐PTFE was ≈24.3 µm (Figure 7f). Notably, PFSA was well impregnated into the CA‐PTFE matrix, forming a three‐layered structure, as indicated by the SEM image of the CA‐PTFE RCM (Figure 7g). The middle layer represents the PFSA‐impregnated PTFE region, and the top and bottom layers correspond to excess PFSA deposited during the impregnation process.

Mercury intrusion porosimetry was used to determine the degree of ionomer impregnation into the reinforcements (Figure 7h). Compared to the commercial ePTFE reinforced RCM (c‐PTFE RCM), which exhibited a porosity of 22.96%, the CA‐PTFE RCM showed a reduced porosity (14.42%), indicating the effective penetration of the PFSA resin into the pores in the reinforcement. Figure 7i illustrates the water uptake properties of the commercial Nafion membranes (NR212), c‐PTFE RCM and CA‐PTFE RCM. The CA‐PTFE RCM demonstrated a higher water uptake of 83.4% than that of the c‐PTFE RCM (73.7%). In contrast, the CA‐PTFE RCM displayed fewer dimensional changes in all directions (Figure 7j) than the NR212 and c‐PTFE RCMs, allowing it to maintain its original shape with greater stability despite the water‐induced swelling of the fully hydrated PFSA ionomers within its matrix.

The proton conductivities of the CA‐PTFE RCMs were higher than those of pristine NR212 and c‐PTFE RCM at all temperatures. This enhancement is attributed to the robust reinforcement effect of CA‐PTFE under hydrated conditions, which suppresses volume expansion and maintains close proximity between the sulfonic acid groups in PFSA and the high density of hydronium ions. Furthermore, the well‐connected ionomer domains in the rectangular channels of the CA‐PTFE support provide more effective proton conduction pathways than the randomly networked, narrow channels in the c‐PTFE membrane. Finally, the CA‐PTFE RCM demonstrated excellent fuel cell performance under both low and high humidity conditions, outperforming currently reported RCMs (Figure 7k). Notably, the hydrogen crossover level remained below 10 mA cm^−2^ for 21 000 humidity cycles, indicating that the developed CA‐PTFE RCM possess the sufficient mechanical durability to meet the DOE technical target for FCEVs.

## 4 Summary and Outlook

This review highlights the potential of IB‐assisted e‐spinning as a versatile and cost‐effective method for the fabrication of biaxially aligned nanofibers. By modifying the E‐field above the substrate with insulating blocks (IBs), this technique enables the precise alignment of e‐spinning jets, offering adaptability to a wide range of materials, including polymers, metals, ceramics, carbons, and composites. In addition, CA‐CNF has been successfully used as a high‐performance filtration material and as both an electrode and a current collector in next‐generation LIBs, effectively fulfilling the dual functions of energy storage and conduction. Furthermore, biaxially aligned polymeric nanofibers in composite membranes have demonstrated improved performance and durability in applications such as flexible aqueous batteries and PEM fuel cells. These examples underscore the potential of IB‐assisted e‐spinning to revolutionize various industries by enabling the development of high‐performance, precisely aligned nanofiber materials.

Despite its potentials, IB‐assisted e‐spinning faces challenges in terms of industrial scalability. Although uniaxial alignment can be achieved through roll‐to‐roll production,^[^ ^35^ ^]^ continuous biaxial alignment on larger scales requires further optimization of the process and formulation parameters. Precise control of the E‐field and production conditions is essential, as the alignment quality may degrade with increased production scales, potentially reducing the degree of fiber organization. Overcoming these challenges is critical for ensuring the scalability and consistency of IB‐assisted e‐spinning. Although it is still in the early stages of development, IB‐assisted e‐spinning shows substantial potential for innovation. Addressing the current challenges will unlock its full capabilities, making it a key technology for next‐generation nanomaterial fabrication. Biaxially aligned nanofibers with tailored morphologies are expected to drive advancements in diverse fields, paving the way for their transformative application in advanced technologies and industries.

## Conflict of Interest

The authors declare no conflicts of interest.
